# Effect of the Hydroalcoholic Extract of *Heracleum persicum* (Golpar) on Folliculogenesis in Female Wistar Rats

**Published:** 2012-06-13

**Authors:** Atefeh Hemati, Mahnaz Azarnia, Mohammad Nabiuni, Ghadireh Mirabolghasemi, Saeed Irian

**Affiliations:** 1. Department of Biology, Faculty of Science, Tarbiat Moallem University of Tehran, Tehran, Iran

**Keywords:** *Heracleum persicum*, Rat, Folliculogenesis, Follicle Stimulating Hormone

## Abstract

**Objective:**

Medicinal plants are widely used throughout the world. Since these plants are known to have minimal side effects, many people embrace them. The golpar plant, scientifically known as *Heracleum persicum (H. persicum)*, is a common Asian and Iranian medicinal plant. The use of golpar is recommended in traditional medicine as a contraceptive medication for females; however, no scientifically documented evidence has been reported. This study investigates the effects of the golpar plant on ovarian tissue and folliculogenesis.

**Materials and Methods:**

In this experimental study, *H. persicum* hydroalcoholic extract (HPHE) was used at 400 mg/kg and 1600 mg/kg doses. Adult female rats were divided into three groups: control, sham, and experimental(I, II). The control group did not receive any injection, the sham group received saline solution, and the experimental group received IP injections of HPHE for 21 days, once every other day, during the sexual cycle. At the end of the injection period, ovarian samples were harvested for histological studies. The FSH assay was performed according to the chemiluminescence immunoassay (CLIA) method. Data were statistically analyzed by the Instat3 program and one-way ANOVA. A p value of <0.05 was considered significant.

**Results:**

In the experimental group the numbers of primordial and primary follicles increased (p <0.001), while the number of preantral and antral follicles decreased (p <0.01). The atretic follicles decreased in the experimental group, but this decrease was not significant. There was no statistical difference in FSH concentration when compared with the control group.

**Conclusion:**

This report gives primary information on the in vivo effects of the HPHE on the ovarian follicles of the female Wistar rat. The results suggest that administration of HPHE may have inhibitory effects on folliculogenesis and cause infertility in females.

## Introduction

Due to the side effects of many chemical drugs, the alternative use of natural medications has greatly increased within the past decade. The therapeutic effects of medicinal plants, which are used as a food-relish in folk medicine, are well documented. *Heracleum persicum (H. persicum)*, commonly known as golpar, is recommended in traditional medicine as a cure for numerous diseases. This plant belongs to the Apiacea (Umbelliferae) family (1).

 Recently, research has shown many benefits of *H. persicum*. Observations in the field of herbal medicine have shown extraordinary anti-inflammatory effects of the root extracts of certain plants from this family (2, 3). Research has shown the presence of furanocoumarin and sphondin compounds in the fruit of this plant (4, 5). In addition, studies on some alkaloids of this plant have noted their anticonvulsant and cytotoxic effects (6, 7). Because of the presence of compounds such as butyrate and octyl acetate, this plant has limited use as a food-relish. In traditional medicine it was observed that use of *H. persicum* during the sexual cycle stops progression of the ovarian phase in females.

 Due to the widespread use of *H. persicum* fruit as a medicinal plant and flavoring agent, and based on Iranian folk medicine's ideas regarding its effect on females, this study aims to evaluate the activities of its hydroalcoholic extract on rats. The purpose of this research is to assess the effects of *H. persicum* on ovarian follicles in adult female rats.

## Materials and Methods

### Plant material and preparation of hydroalcoholic extract

*H. persicum* plants were collected from the suburbs of Shemiran, in northern Tehran (Iran) at the end of August 2009. Samples were pressed and dried according to herbarium techniques. The fruits of *H. persicum* were dried and powdered under natural conditions. For preparation of the hydroalcoholic extract, 200 g of the fruit was powdered, air-dried, and macerated with 1500 ml of EtOH–H_2_O (1:1) for 48 hours. The combined extract was filtered and evaporated to dryness for 5-6 hours.

### Animals

All animal experiments were carried out according to the guidelines of the Iranian Council for Use and Care of Animals and approved by the Animal Research Ethical Committee of Tarbiat Moallem University of Tehran. Adult female Wistar rats (n=24, 4-6 weeks old) weighing 180-220 g were used in this study. Rats were allowed free access to food and water at all times. They were maintained in groups of six, one group per standard cage, under a 12 hour light-dark cycle.

### Administration

Adult female rats were divided into three groups: control, sham, experimental(I and II). Each group included six rats. Rats in experimental group I received IP injections of 400 mg/kg *H. persicum* extract; those in experimental group II received 1600 mg/kg, IP. In the experimental groups, injections of *H. persicum* extract were administered over a period of 21 days, once every other day during the sexual cycle. In the sham group, saline was injected as the extract solvent. The control group received neither solvent nor extract.

 To study the effects of the *H. persicum* hydroalcoholic extract (HPHE) during the sexual cycle, vaginal smears of the adult female rats were performed and prepared for histological studies. Based on the morphology of the vaginal epithelium, various stages of the estrous cycle were determined. Rats with regular sexual cycles were selected and IP injections of different dosages of the extract were made once every other day throughout the sexual cycle.

### Hormonal assay

The animals were anesthetized with ether; blood samples were collected directly from their hearts and centrifuged at 2000-3000 rpm for 15 minutes at 4ºC. Serum samples were stored at -20ºC until assayed for FSH. Serum concentrations of FSH were measured by the chemiluminescence immunoassay (CLIA) method.

### Histological analysis

After 21 days the tissues were removed. The left ovary was removed and placed in formalin fixative for 20-24 hours. Fixed tissue samples were placed in ascending concentrations of alcohol and embedded in paraffin. Slices of tissue, 5-7 µm thick, were prepared and stained with hematoxylin and eosin (H&E), and then monitored and evaluated with a light microscope. To study folliculogenesis all tissue blocks were serially sliced. The first and every fifth section were selected (10 sections total) and placed on 10 different slides. In total, about 50 sections from each ovary were studied. Follicle identification was based on the detection of a nucleus. The numbers of follicles (primordial, primary, etc.) were counted. Follicle recognition criterion on the slides was based on the type of epithelial cells surrounding them. For example, primordial follicles have squamulose cells whereas primary follicles are surrounded by cuboidal cells. The numbers of follicles per slide were randomly counted in triplet fields of a microscopic view.

### 

Statistical analysis Means were compared by one-way analysis of variance (ANOVA) using the Instat3 program. p<0.05 was considered significant. We used the Tukey post hoc test for analysis.

## Results

**Fig 1:**
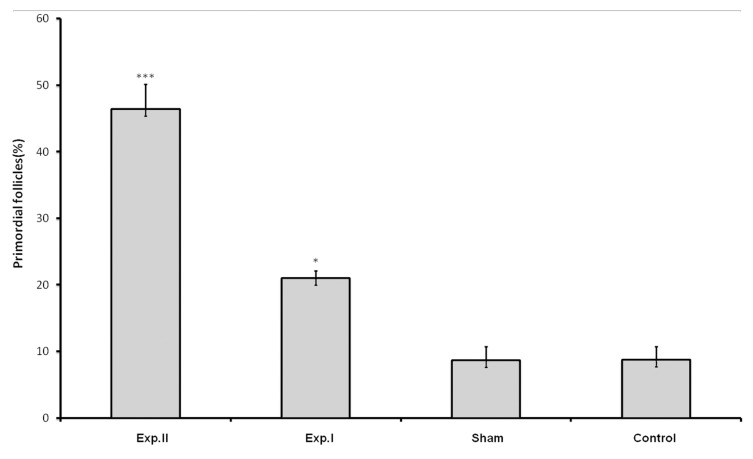
The percentage of primordial follicles in animals treated with HPHE in experimental [exp. I (400mg/kg) and exp. II (1600 mg/kg)], Sham, and untreated (Control) animals. *** p<0.001, * p<0.05.

**Fig 2:**
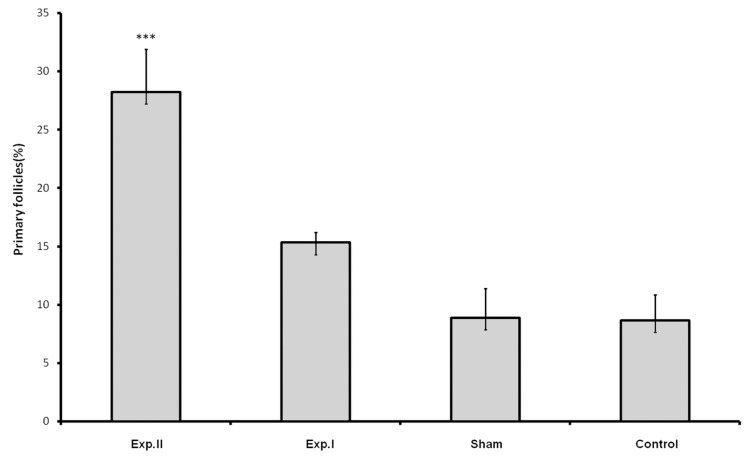
The percentage of primary follicles in animals treated with HPHE in experimental [exp. I (400 mg/kg) and exp. II (1600 mg/kg)], Sham, and untreated (Control) animals. *** p<0.001.

**Fig 3:**
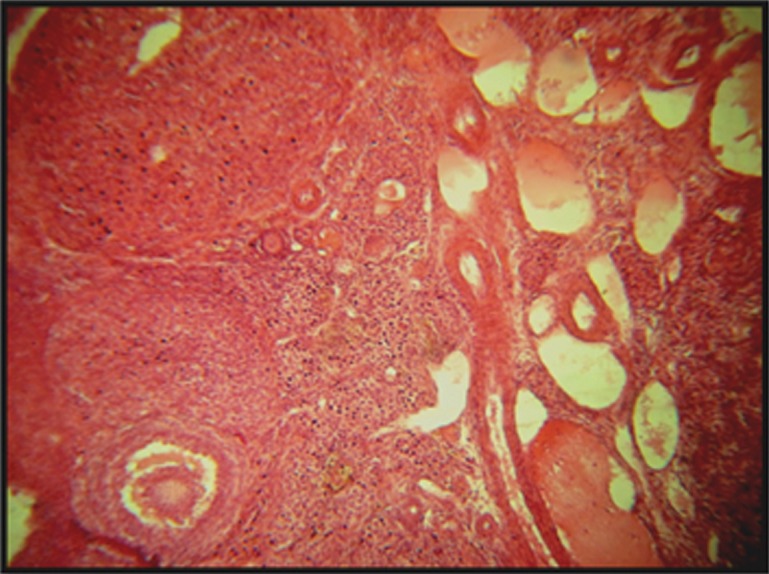
Increased primordial follicles in rat ovaries treated with H. persicum hydroalcoholic extract (HPHE) at 400 and 1600 mg/kg doses. ×200.

**Fig 4:**
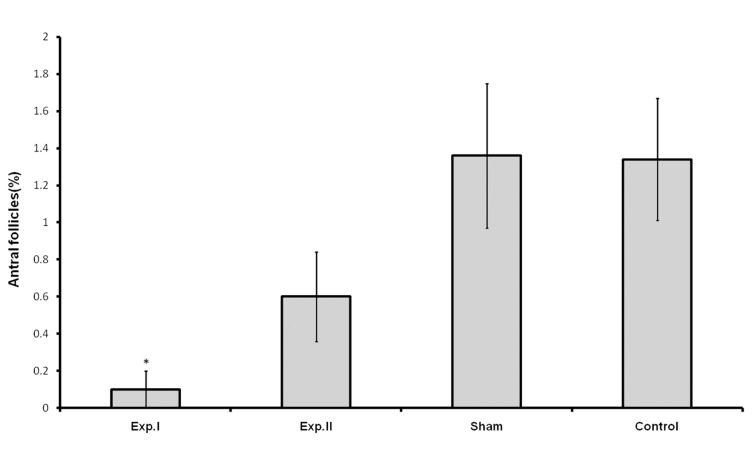
The percentage of antral follicles in animals treated with HPHE in experimental [exp. I (400mg/kg) and exp. II (1600 mg/kg)], Sham, and untreated (Control) animals. *p<0.05.

**Fig 5:**
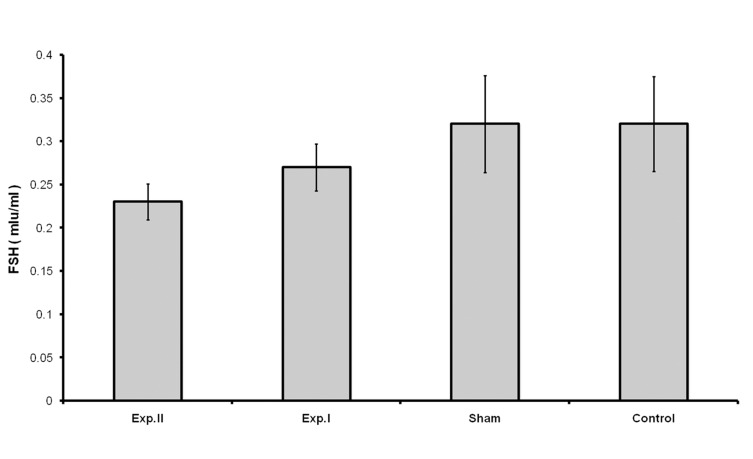
Plasma levels of FSH in animals treated with H. persicum extract in experimental [exp. I (400mg/kg), exp. II (1600 mg/kg)], Sham, and untreated (Control) groups. The plasma levels of FSH were determined by CLIA; p>0.05.

Treatment with dosages of 400 and 1600 mg/kg of HPHE significantly increased the number of primordial follicles (p<0.05 for 400 mg/kg; p<0.001 for 1600 mg/kg; Table 1, Figs 1, 3). This increase was also observed in the number of primary follicles, however it was significant only in the 1600 mg/kg group (p<0.001, Table 1, Fig 2). Treatment with 400 and 1600 mg/kg dosages of the extract decreased the number of preantral and antral follicles, however, this decrease was significant only in the 400 mg/kg group (p<0.05, Table 1, Fig 4). Different dosages of the extract slightly increased the number of atretic follicles; a greater increase was observed at 1600 mg/kg (p>0.05; Table 1). Finally, treatment with both dosages of HPHE caused a slight decrease in the amount FSH, which was not significant (Table 1, Fig 5).

**Table 1:** Mean ± SE values of primordial, primary, preantral, antral, and atretic follicles, and the amount of FSH in experimental (Exp. I and Exp. II), control, and sham groups


	Exp. I	Exp. II	Control	Sham

Primordial follicles	21 ± 1.09	46.4 ± 3.75	8.72 ± 2.02	8.66 ± 2.08
Primary follicles	15.31 ± .09	28.21 ± 3.68	8.62 ± 2.24	8.86 ± 2.53
Preantral follicles	2 ± 0.54	2.8 ± 0.96	3.54 ± 0.62	3.47 ± 0.61
Antral follicles	0.12 ± 0.10	0.60 ± 0.24	1.34 ± 0.33	1.36 ± 0.39
Atretic follicles	6.41 ± 0.81	9.4 ± 1.83	4.71 ± 1.41	4.82 ± 1.38
FSH	0.27 ± 0.02	0.23 ± 0.02	0.32 ± 0.05	0.32 ± 0.05


## Discussion

HPHE in the first stages of folliculogenesis strongly increased the number of primordial follicles. This increase was more pronounced at the 1600 mg/kg dose of the extract in that HPHE acted as a stimulant, causing progression of folliculogenesis to the stage of primary follicle formation. The increase in the number of primary follicles more pronounced at a dosage of 1600 mg/kg than at the 400 mg/kg dosage. Maximum follicular growth and maturity occurred at the stage of preantral and antral follicle formation. However, at the next stage of folliculogenesis HPHE caused a decrease in the number of growing follicles. Here the extract appeared to act as a repressing agent, blocking the progression of folliculogenesis in a way that a significant decrease was observed in the number of antral follicles at the 400 mg/kg dosage. The extract also caused an increase in the number of atretic follicles, which confirmed the repressing effect of the extract on the natural growth of follicles, which seems reasonable considering the slight decrease in the level of FSH.

Compounds such as sphondin affect the pathway of inflammation. Sphondin represses the expression of IL-1β-induced cyclooxygenase, and through this pathway it plays an important role in inflammation (8, 9). The process that occurs in mammalian ovaries is similar to inflammation (10, 11). Sphondin, one of the compounds present in HPHE, may repress folliculogenesis through this pathway (12, 13). The antioxidant compounds present in this plant could have a direct effect on inflammation, and exert their effect by blocking the production of free radicals which play a key role in inflammation (14, 15).

The hydroalcoholic extract of *H. persicum* can repress natural follicular growth and maturation, preventing the progression of folliculogenesis. This repressing effect is more severe at the 400 mg/kg dosage. As this repressing property exerts its effect at a specific stage of folliculogenesis in the initial stages of the cycle, it causes an increase in the number of primordial and primary follicles. Thus it is possible that the inhibitory effect of this plant is partial, and concrete evidence on proving this effect would require further investigation.

## Conclusion

This study shows, for the first time, that the theory behind using H. persicum as a contraceptive and a repressor of the sexual cycle in women, as mentioned in traditional medicine, might be true. However concrete evidence for introducing this plant as an effective drug in the field of sterility and fertility would need further investigation.
